# The Interrelationship between Leukotriene B4 and Leukotriene-A4-Hydrolase in Collagen/Adjuvant-Induced Arthritis in Rats

**DOI:** 10.1155/2014/730421

**Published:** 2014-02-20

**Authors:** Mariana Trivilin Mendes, Paulo Flavio Silveira

**Affiliations:** ^1^Laboratory of Pharmacology, Unit of Translational Endocrine Physiology and Pharmacology, Instituto Butantan, Avenida Vital Brasil, 1500, 05503-900 São Paulo, SP, Brazil; ^2^Department of Physiology, Instituto de Biociencias, Universidade de São Paulo, Rua do Matão, Travessa 14, No. 321, 05508-090 São Paulo, SP, Brazil

## Abstract

This study aimed to check the involvement of lipid mediator leukotriene (LT) B4 and the activity of LTA4 hydrolase (LTA4H) in the development of arthritis induced in rats by collagen and adjuvant (CIA). High-performance liquid chromatography (HPLC) and enzyme immunoassay (EIA) were used for measurements of LTB4 and LTA4H in plasma, synovial fluid (SF), soluble (SO), and solubilized membrane-bound fraction (MB) from synovial tissue (ST) and peripheral blood mononuclear cells (PBMCs) of CIA-arthritic and CIA-resistant. EIA process is simple, clean, and rapid and offered advantages over HPLC, showing that in SF and MB-PBMCs of CIA-arthritic and CIA-resistant, and in MB-ST of CIA-resistant, LTB4 and LTA4H were altered in parallel and were positively related. In the plasma and SO-ST and SO-PBMCs of CIA-arthritic and CIA-resistant, and in MB-ST of CIA-arthritic, this pattern was not found. The primordial role played by LTA4H in the biosynthesis of LTB4 was confirmed together with the existence of alternative steps that regulate LTB4 without participation of LTA4H. The involvement of compartmentalized and coupled changes of LTB4 and LTA4H in the resistance and development of arthritis in CIA model was demonstrated for the first time.

## 1. Introduction

The etiology and the mechanisms of rheumatoid arthritis chronicity [1–4] are still poorly understood. This disease has been extensively studied in animal models in that is induced by administration of antigens and/or adjuvants [5], among them, type II collagen (CII) and Freund's adjuvant [6–8] are the most widespread (CIA model). The main known features that are common for CIA model and rheumatoid arthritis are synovitis, progressive pannus formation, marginal erosion of bone, and cartilage destruction [6–9].

The involvement of the leukotriene (LT) B4 (acid 5[S],12[R]-dihydroxy-6.14*cis*-8,10-*trans*eicosatetraenoic) in the development of arthritis is an attractive hypothesis. LTB4 is a potent proinflammatory lipid mediator synthesized by cells of the immune system and stimulates the production of several cytokines [10]. LT synthetic route is known to have three critical factors. The first is related to the activity of 5-lipoxygenase (5-LO) (key enzyme in the LT biosynthesis), which incorporates a hydroperoxy group at the 5-carbon from arachidonic acid, forming the 5-hydroperoxyeicosatetraenoic acid, and then dehydrates this molecule at 10-carbon to form LTA4. The LTA4 can be converted in LTC4 by its conjugation with glutathione. Thus, the second critical factor is related to LTC4 synthase, which generates LTC4. Subsequently, gamma-glutamyl transpeptidase generates LTD4 from LTC4. LTD4 is then cleaved by dipeptidases to form LTE4. Finally, the third factor is related to the activity of LTA4 hydrolase (LTA4H), which generates LTB4 by hydrolysis of LTA4 [10, 11]. LTA4H activity is known to be exerted by two bifunctional zinc metalloenzymes, EC 3.3.2.6, the canonical LTA4H [12], and EC 3.4.11.6 (basic aminopeptidase) [13, 14]. Little is known about the pathophysiological role of LTB4 and even more of LTA4H activity due to LTA4 lability and important doubts about the efficiency of the available methodologies to quantify this activity. Only very recently an efficient synthetic substrate for LTA4H was reported [15]; however, this substrate has not yet been used to evaluate this activity in any disease. LTB4 has been measured by high-performance liquid chromatography (HPLC), since it allows the use of standard (prostaglandin B1 or B2) with retention time (RT) similar to the LTB4, but with lower cost and lability [16, 17], and by enzyme immunoassay (EIA) using monoclonal antibodies against LTB4 [18]. However, the relative effectiveness of these two methods using samples obtained under different pathophysiological conditions and handling is still unknown.

This study aimed to check the involvement of LTB4 content and LTA4H activity, measured by HPLC and EIA, on the development of experimental arthritis in rats.

## 2. Materials and Methods

### 2.1. Chemicals and Reagents

LTB4 EIA kit, using monoclonal antibody produced in mice against LTB4 from rabbit, and LTB4 were from Cayman Chemical (USA). CII from chicken, dimethyl sulfoxide, Freund's incomplete adjuvant, HEPES buffer, LTA4, Turk's fluid, Trypan, and NaCl were from Sigma-Aldrich (USA). Acetic acid was from Labsynth Produtos para Laboratorio Ltd. (Brazil). Glycerol was from USB Corporation (USA). Ketamine 5% (Vetanarcol) was from König do Brasil (Brazil). Percoll (*ρ* = 1.077 mg/mL) was from GE Healthcare (USA). Sodium heparin 25,000 UI/5 mL (Liquemine) was from Roche (Brazil). Xylazine 2.3% (Anasedan) was from Sespo Ind. Co., Ltd., Vetbrands Division (Brazil). All other chemicals and reagents were of analytical grade and purchased from Merck KGaA (Germany).

### 2.2. Animals and Treatments

Adult male Wistar rats, weighing 160–180 g and maintained in polyethylene cages with food and tap water *ad libitum* in a container (Alesco Ind. Co., Ltd., Brazil), with controlled temperature of 25 °C, relative humidity of 65.3 ± 0.9%, and 12 h : 12 h photoperiod light : dark (lights on at 6:00 am), were subjected to the following procedures approved by the Ethics Committee on Animal Use of Butantan Institute (682/09). Based on Cremer [19] method, modified by Mendes et al. [7], the animals were injected with CII from chicken dissolved in 0.01 M acetic acid and emulsified in equal volume of Freund's incomplete adjuvant (prepared at 4 °C just before use), via a single intradermal dose of 0.4 mg/0.2 mL/animal, into the proximal one-third of the tail (induced animals), or with 0.9% NaCl at the same scheme of administration (sham induction). All animals that receive the emulsion or saline were previously anesthetized with a solution of ketamine (3.75%) and xylazine (0.5%) at a dose of 0.2 mL/100 g body mass, via intraperitoneal (ip). All these procedures mentioned above, as well as the evaluation of edema, erythema, and cyanosis and the collection of samples were carried out in the morning.

### 2.3. Macroscopic Assessment of Arthritis and Sample Collection

On 41st day after treatments, the animals were anesthetized using the same scheme specified above. Then, erythema and cyanosis were observed, and the dorsal-plantar thickness of the hind paws in the region of the metatarsus was quantified with a micrometer (Mitutoyo do Brasil, Brazil). Both paws were measured and mean thickness for each animal was calculated. The following experimental groups were formed based on previously described criteria [7, 8]: control (all animals submitted to sham induction); arthritic (induced animals with hind paw thickness > 5.7 mm that also present erythema and cyanosis); and resistant (induced animals without erythema and cyanosis and with hind paw thickness similar to control). These animals were then used for sample collection and were subsequently euthanized. Blood withdrawal was from the left ventricle with heparinized syringes and used to obtain peripheral blood mononuclear cells (PBMCs), or submitted to centrifugation (at 200 ×g for 10 min at 4 °C, centrifuge model CR31, Jouan Inc., USA) to obtain plasma. The synovial fluid (SF) and tissue (ST) were subsequently removed from both knees of each animal as follows: 200 *μ*L of 0.9% NaCl was injected intra-articularly into each knee with an ultrafine needle (0.45 × 13 mm) and aspirated with a syringe and, after such washing, the ST was excised together with the connective tissue of the joint capsule.

### 2.4. Obtaining Peripheral Blood Mononuclear Cells (PBMCs)

According to the method of Grage-Griebenow et al. [20], heparinized blood was carefully layered on Percoll (density = 1.077 g/mL) in PBS (56%) at a proportion of 5 : 3 (v/v) and subsequently centrifuged (1000 ×g for 40 min at 25 °C). The layer containing the PBMCs was then removed from the tube and transferred to microtubes to be immediately used.

20 *μ*L aliquots of PBMCs suspension were diluted with Turk's fluid (1 : 20, v/v). The cell viability was assessed using 40 *μ*L aliquots of this suspension diluted in equal volume of Trypan. Cell counting was performed in a Neubauer chamber (Inlab, Brazil) under optical microscopy (microscope model Eclipse E600, Nikon, Japan).

### 2.5. Fractionation of ST and PBMCs

As previously described by Mendes et al. [7], the ST from both knees of each animal was homogenized in 10 mM Tris-HCl buffer, pH 7.4 (0.1 g tissue/3.0 mL) for 3 min at 15,000 rpm (homogenizer model Polytron-Aggregate, Kinematica, Switzerland). PBMCs homogenates were sonicated in 10 mM Tris–HCl, pH 7.4 (3.0 × 10^6^ cells/mL), for 10 sec at amplitude level of 40 *μ*m at a constant frequency of 20 kHz (sonicator model Sonics VibraCell, Sonics & Materials Inc., USA). These samples were then ultracentrifuged at 100,000 ×g for 35 min (ultracentrifuge model CP60E, Hitachi, Japan). The resulting supernatants correspond to soluble (SO) fraction. The resulting pellets were washed twice with the same buffer and ultracentrifuged at 100,000 ×g for 35 min, to assure the complete removal of SO. The pellet was homogenized for 3 min at 800 rpm (homogenizer model TE 099, Tecnal, Brazil) with the same volume of the same buffer plus Triton X-100 (0.1%) and ultracentrifuged again (100,000 ×g for 35 min). The resulting supernatants correspond to solubilized membrane-bound (MB) fraction. All procedures were carried out at 4°C. The efficiency of this fractionation in both materials was previously demonstrated using lactate dehydrogenase activity as a marker [7, 8]. During the fractionation procedures, no protease inhibitor was used and thus some degree of loss of LTA4H activity as a function of its proteolytic degradation could not be discarded.

### 2.6. LTB4 and LTA4H Measurements

#### 2.6.1. Incubation of Samples with or without LTA4

Based on the methodology of Liang et al. [21], 1 *μ*L of LTA4 solution (100 *μ*g/mL) was diluted in 1499 *μ*L of 50 mM HEPES buffer, pH 7.5, containing 0.0625% glycerol and 1% dimethyl sulfoxide. Alternatively, 1 *μ*L of LTA4 solution was substituted by 1 *μ*L 0.9% NaCl in this mixture. 50 *μ*L of plasma, SF, and SO and MB from ST and PBMCs and 250 *μ*L of buffer solution mentioned above containing or not LTA4 (LTA4 final concentration = 166.66 nM) were pipetted into each microplate well (96-well flat bottom microplates, Corning, USA) and then incubated (25°C) under orbital shaking (250 rpm) (model PST-60 HL plus, Biosan, Brazil) for 10 min. Thus, 10 *μ*L of each incubated mixture were transferred to a microtube containing 190 *μ*L of ice-cold assay buffer (EIA buffer) from EIA kit for LTB4.

#### 2.6.2. Enzyme Immunoassay (EIA)

Absorbance at 412 nm of each sample obtained as described above and of the peaks corresponding to the RT of LTB4 that were obtained from HPLC (see below) was read against two HEPES buffer solutions, with or without LTA4; both considered a “blank” of each kind of incubation, and also against the “blank” supplied with the kit.

#### 2.6.3. HPLC

To perform the extraction of LTB4, 290 *μ*L samples (incubated with or without LTA4) and synthetic standard LTB4 (100 *μ*g/mL) diluted in methanol : tetrahydrofuran : acetonitrile : deionized H_2_O (10 : 10 : 30 : 50, v/v) (solution A) were previously acidified with 1 M formic acid (pH = 3.5) and applied to Sep-Pak C18 microcolumns (Waters, Ireland), previously equilibrated, sequentially, with 5 mL of methanol and 5 mL of deionized water. After sample injection, the column was washed with 5 mL water and 5 mL hexane and kept for 5 min at 25°C. Subsequently, the LTB4-containing samples were eluted with 5 mL ethyl acetate in 1% methanol (99 : 1, v/v), and these eluates were aliquoted and dried in SpeedVac (model SC 100, Savant Instruments Inc., USA) [22]. Dried eluates were stored at −20°C until analyses by HPLC system (LC-20AT, CBM CBM-20A, DGU-20A_3_, Prominence, Shimadzu, Japan). HPLC was performed using an analytical column (4.6 mm diameter × 25 cm long, particle size 5 mm) reverse phase C18 (Shimadzu, Japan) and a spectrophotometer UV/VIS SPD-20A detector (model Prominence, Shimadzu). Absorbance was monitored at 270 nm. Dried eluates were dissolved in 130 *μ*L solution A and 40 *μ*L was then applied in the column previously equilibrated in solution A. Methanol : acetonitrile : acetic acid : deionized H_2_O (30 : 30 : 0.01 : 40 v/v) was used as mobile phase in an isocratic mode of elution at a flow rate of 0.8 mL/min for 20 min [17]. Fractions corresponding to LTB4 peak were manually collected. The peaks were identified by comparing their RTs with that of synthetic standard LTB4. The amount of LTB4 in the samples was estimated by comparing the areas of the peaks of the sample and the standard. The peak area was calculated automatically by the HPLC system using the LC solution software package (Lab Solution, Shimadzu) as ∫AU × *dt*, being AU = absorbance unit = optical density at 270 nm enclosed by the initial and final baselines of the peak curve, and *dt* = time course, in seconds, between the initial and final baselines of the peak curve. The same percentage of recovery was considered, since the sample and the standard were submitted to the same conditions of Sep-Pak C18 microcolumn extraction and HPLC procedures.

#### 2.6.4. Catalytic Activity

The values of the blanks were subtracted and the relative absorbance was converted to ng of LTB4 formed in 1 min of incubation per 1 mL of sample, by an interpolation in a correspondent standard curve (EIA or HPLC). The values of LTB4 formed in each samples incubated without LTA4 (endogenous LTB4) were subtracted from the values of LTB4 in the same samples incubated with LTA4, thus representing the value of LTB4 formed *in vitro*. The rate of this catalysis was evaluated by the concentration of LTB4 produced per unit time and thus expressed as LTA4H unit (U), which was defined as the abundance of LTA4H activity in 1 mL sample that catalyzes the formation of 1 picomole of LTB4 per minute.

### 2.7. Statistical Analysis

The data are shown as mean ± standard error of the mean (SEM) and were analyzed statistically using the GraphPad InStat software package. Regression analyses were performed to obtain standard curves of LTB4 and correlation between LTB4 values obtained by EIA and HPLC. To compare values among the control, CIA-arthritic, and CIA-resistant groups, one-way analysis of variance (ANOVA) was performed, followed by Student-Newman-Keuls multiple comparisons test when differences were detected. In all the calculations a minimum critical level of *P* < 0.05 was set.

## 3. Results

### 3.1. Classification of Experimental Animals Based on the Formation of Edema, Erythema, and Cyanosis

The swelling (in mm; *n* = number of animals; analysis of variance ANOVA *P* < 0.0001; Student-Newman-Keuls multiple comparisons test; *P* < 0.001), erythema, and cyanosis were the main macroscopic characteristics of the hind paws of arthritic animals. Severe swelling (6.0 ± 0.04, *n* = 10) was found in 60% of animals treated with CII and adjuvant. Compared with control (4.7 ± 0.05, *n* = 10), 30% of the animals treated with CII and adjuvant showed no erythema, cyanosis, or edema (4.8 ± 0.30, *n* = 10). These data matches the differential detection of serum tumor necrosis factor alpha and histopathological alterations of tibiotarsal joint in the CII and adjuvant treated animals that develop severe edema (CIA-arthritic), which can be thus confidentially distinguished from CIA-resistant (without edema) [7, 8]. Based on macroscopic classification of edema formation, preconized by Erlandsson Harris et al. [23], all arthritic animals selected here had the maximum score in hind paws. Additionally, 10% of CII and adjuvant treated animals were discarded because they did not reach this level of arthritis.

### 3.2. Comparison of Two Strategies for Measurements of LTB4 and LTA4H

**Figure 1:**
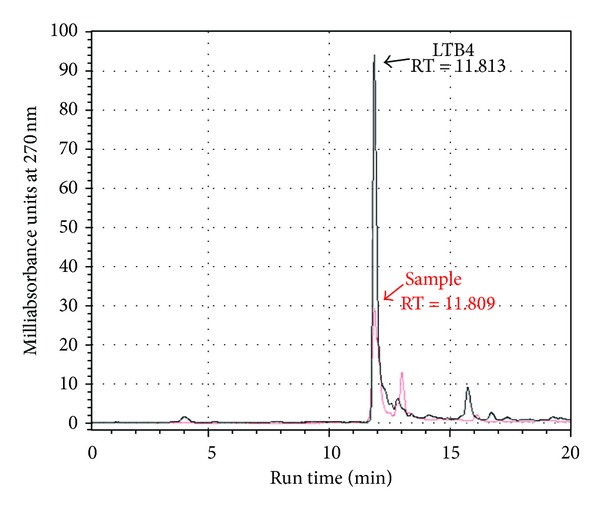
HPLC chromatograms of 0.4 *μ*M standard LTB4 (in black) and synovial fluid from arthritic incubated with LTA4 (in red). The same profile was obtained with the other samples from other animal groups under study.

**Figure 2:**
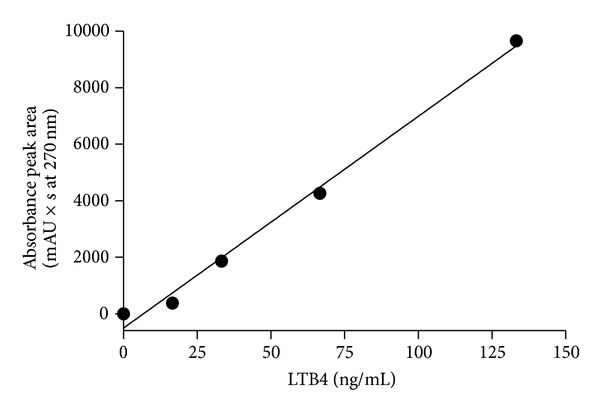
Standard curve between known concentrations of standard LTB4 and corresponding HPLC peak areas; slope = 74.96 ± 3.83, *r* ^2^ = 0.9922, *P* = 0.0003.

**Figure 3:**
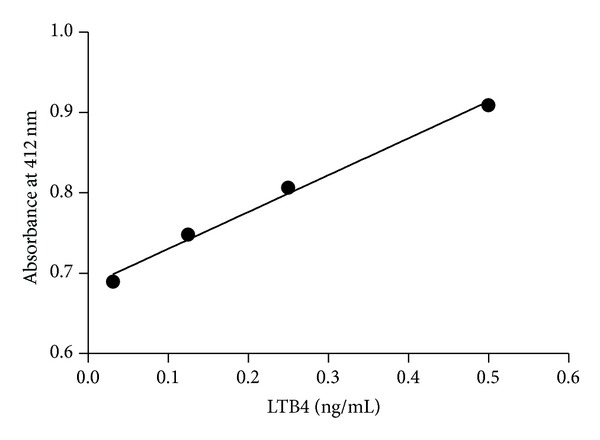
Standard curve between known concentrations of standard LTB4 and absorbance values in EIA; slope = 0.4588 ± 0.0285, *r* ^2^ = 0.9923, *P* = 0.0038.

**Figure 4:**
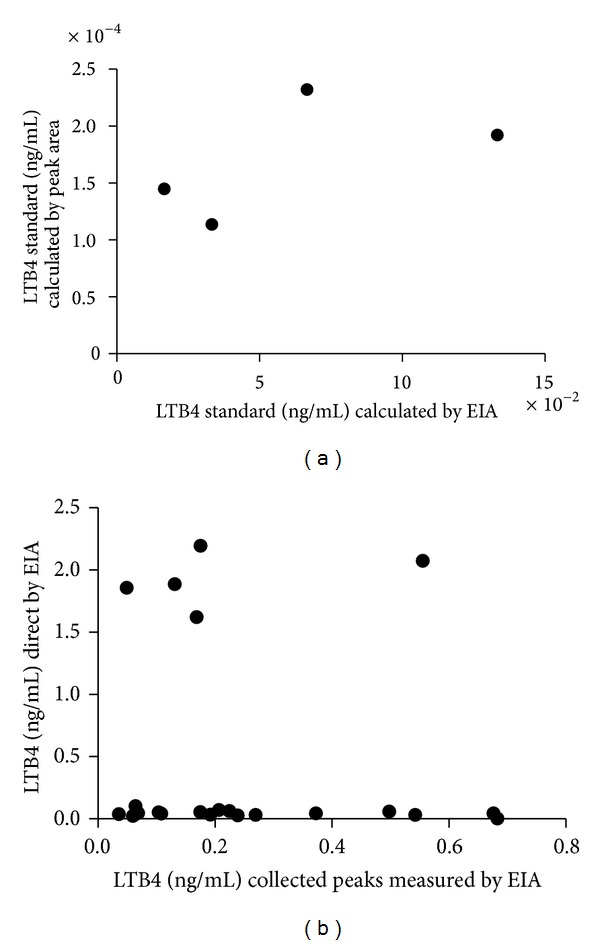
Comparative regression analysis on (a) concentrations of standard LTB4 measured by EIA in the resulting peaks after HPLC of this standard (*n* = 4); slope = 0.0006 ± 0.0006, *r* ^2^ = 0.3280, and *P* = 0.4273 and on (b) concentrations of LTB4 measured by EIA in the resulting peaks after HPLC of samples and directly in these same samples without further processing (*n* = 22); slope = −0.3592 ± 0.8776, *r* ^2^ = 0.0083, and *P* = 0.6867.

**Figure 5:**
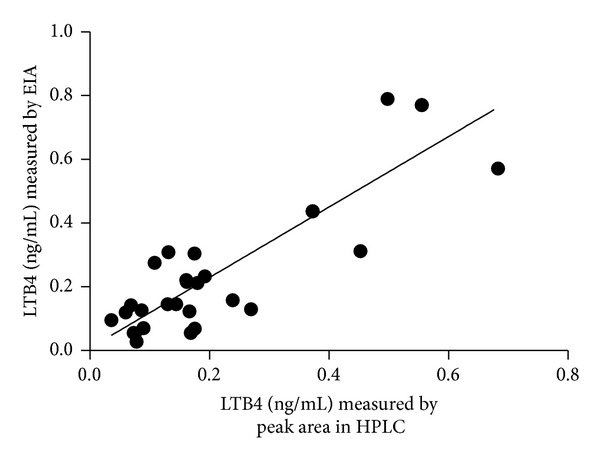
Linear regression between the LTB4 content in all samples under study (*n* = 36), measured by peak area in HPLC and by EIA; slope = 1.106 ± 0.1207, *r* ^2^ = 0.7566, and *P* < 0.0001.

Figure 1 illustrates the chromatographic profiles of standard LTB4 (in red) and samples (in black). Fractions corresponding to LTB4 peak (RT = 11.5–13.5 min, total volume = 1.6 mL) were pooled, dried, and stored at −80°C.  Figure 2 illustrates the standard curve of peak areas obtained with known concentrations of LTB4 applied into the HPLC.  Figure 3 illustrates the standard curve of LTB4 in EIA.  Figure 4(a) shows the regression analysis between different known concentrations of standard LTB4 and those measured by EIA in the resulting peaks after HPLC of standard LTB4, showing the absence of correlation.  Figure 4(b) shows the regression analysis between LTB4 measured by EIA in the resulting peaks after HPLC of samples and directly in these same samples without further processing, confirming the lack of correlation observed in Figure 4(a). Neither theoretical LTB4 concentrations in the HPLC peaks and their measurements by EIA nor the LTB4 of the samples measured by EIA before HPLC and in the HPLC peaks is correlated (Figures 4(a) and 4(b)), but Figure 5 shows that there is a consistent linear correlation between the concentrations of LTB4 obtained from the calculation of peak areas and the concentrations of LTB4 obtained by EIA in all samples under study. In general, as expected, the concentration values of LTB4 obtained by calculation of peak areas and directly by EIA are equivalent in the samples incubated or not with LTA4 (Figure 5).

### 3.3. Evaluating LTB4 and LTA4H in Control, CIA-Arthritic, and CIA-Resistant

The values obtained by EIA for expressing the LTB4 content (Table 1) and for the calculation of LTA4H activity (Table 2) were used throughout the remainder of this study due to their easier running and higher accuracy than HPLC. Table 1 shows the values of EIA without incubation with LTA4, which reflect the endogenous LTB4 content of samples. These values compared with control are higher in SF and MB-ST and lower in SO-ST and in MB-PBMCs of CIA-arthritic and CIA-resistant, and lower in SO-PBMCs from CIA-resistant. Table 2 shows *in vitro *LTA4H activity in plasma, SF, SO-ST, MB-ST, SO-PBMCs, and MB-PBMCs, calculated by subtraction of values of each sample without incubation (see Table 1) from those after incubation with LTA4 (data not shown), obtained by direct measurement in EIA. Compared with control, the LTA4H activity is higher in SF and SO-PBMCs and lower in MB-PBMCs of CIA-arthritic and CIA-resistant, and higher in MB-ST of CIA-resistant. Comparing LTB4 content (Table 1) and LTA4H activity (Table 2) between CIA-arthritic and CIA-resistant, differences were detected in the first parameter in MB-ST and SO-PBMCs (higher in CIA-arthritic) and in the second parameter in SF, MB-ST, and SO-PBMCs (higher in CIA-resistant) (Table 3). Table 3 illustrates altered LTA4H activity levels and LTB4 content in CIA-arthritic and CIA-resistant compared with control.

## 4. Discussion

In this study, we showed that the correlations between LTB4 and LTA4H have significance on resistance and development of arthritis in CIA model.

Arthritis and resistance in CIA model, at the beginning of the development of the disease, can be evidenced, respectively, by the presence of erythema, cyanosis and severe edema in both hind legs, as well as by typical histopathological aspects of the tibiotarsal joint (arthritic), or by the complete absence of these characteristics (resistant) [7, 8]. These characteristics were those considered for grouping the animals.

Measurement of LTB4 by HPLC, as described by Rudberg et al. [17], is methodologically adapted, simplified, and revalidated in the present study. LTB4 quantification by EIA provides values with a high degree of correlation with those of peak areas obtained by HPLC. Compared with HPLC, the EIA methodology does not need cleaning steps (removal of interfering compounds), thereby simplifying the analysis and shortening its time course, besides its high specificity, low cost, and possibility to use small samples. One disadvantage is that the EIA kits have a limited half-life. It is also noteworthy that EIA has higher sensitivity than HPLC. Furthermore, comparing the LTB4 values obtained in each one of the methods, EIA values have more consistent statistical differences among the groups under study than those obtained by HPLC. Obviously, this feature can be attributed to inherent procedures of HPLC methodology (Sep-Pak column extraction to minimize impurities and drying, for example). HPLC peaks of the standard LTB4 or samples containing LTB4 when measured by EIA do not correlate with values of LTB4 measured by EIA or by peak areas. Thus, the HPLC procedure before EIA can be able to alter the LTB4 antibody binding. Given these results, the present study adopted LTB4 values obtained by EIA to calculate the values of LTA4H activity and to compare these values among experimental groups.

Endogenous LTB4 preexisting *ex vivo* and after *in vitro* incubation with LTA4 was assessed. During this incubation, the LTB4 content preexisting *ex vivo* is added to that formed *in vitro* under the action of LTA4H in such samples. Therefore, a simple subtraction of the preexisting LTB4 from the total LTB4 formed after the incubation is sufficient to estimate the LTA4H activity in the samples. At this point, it was noteworthy that LTA4H activity is detected in the MB-ST and MB-PBMCs. At our knowledge, there is only one previous report (in hepatocytes) about the existence of membrane-bound LTA4H activity [24]. In this way, the pathophysiological significance of this finding requires further studies, in particular an evaluation by immunoblot. Regarding possible supposition about this finding, it is suggestive that, although 5-LO has been originally purified as a cytosolic protein, now it is also known to be translocated to the nuclear envelope [25] and to lipid bodies of eosinophils, after stimulation [26]. Cytosolic phospholipase A2 (cPLA2) is also translocated to the nuclear envelope [27], besides that COX-1 and COX-2 exist in the nuclear membrane then the nuclear membrane is commonly accepted as the main site of eicosanoids production. It is also known that the expression of 5-LO is restricted to B cells, macrophages, monocytes, mast cells, neutrophils, neurons, eosinophils, and dendritic cells [28, 29], but all cell types are potential sources of LTB4, because the LTA4H is ubiquitously expressed, and the excess of LTA4 generated by those cells expressing 5-LO can diffuse it to nearby cells that do not have significant 5-LO activity and, therefore, also lack the ability to generate the substrate LTA4 [29, 30]. Then, these cells expressing LTA4H, and very low 5-LO, can also convert LTA4 into LTB4, in a mechanism known as transcellular biosynthesis [31]. Neutrophils, monocytes, lymphocytes, and erythrocytes are rich sources of LTA4H. In contrast, eosinophils have low levels, while basophils and platelets practically lack this enzyme [30]. The molecular mechanism and the domains of 5-LO required for translocation have been intensively studied [29]. In contrast to the 5-LO, there are limited information about the possible activation and translocation of LTA4H. It is believed that under physiological conditions, LTB4 is produced specially by the leukocytes. However, the production of LTB4 is increased in leukocytes and other cells during inflammation [29, 30], fact which has been explained by transcellular biosynthesis [29, 30, 32]. This phenomenon is demonstrated to be promoted *in vitro* by cell-cell interactions and involved in the formation of leukotrienes in monocytes, lymphocytes [33], neutrophils, endothelial cells, erythrocytes, platelets, and human bronchoalveolar lavage fluid [34–37].

**Figure 6:**
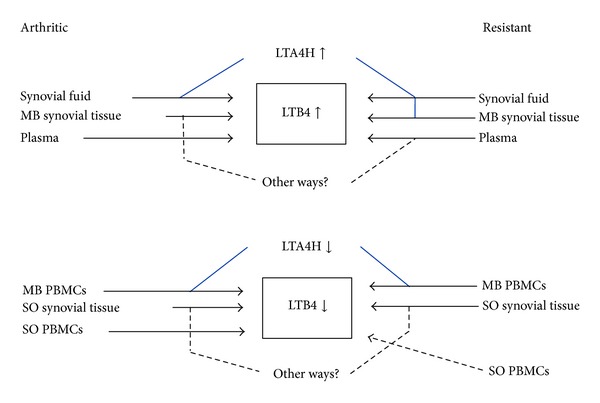
Changes in LTB4 levels and LTA4H activity in CIA-arthritic and CIA-resistant rats. Arrows ↑ and ↓ indicate, respectively, increased or decreased values relatively to healthy control. Blue solid line indicates the situation in which changes in LTB4 content and LTA4H activity are positively related. Black dotted line indicates the situation in which changes in LTB4 content and LTA4H activity are not positively related. It is noteworthy that changes in LTB4 content and LTA4H activity in all samples allow to distinguish between CIA-arthritic and CIA-resistant only when compared quantitatively (Tables 1–3).

LTA4H has been reported to suffer suicide inactivation when exposed to LTA4 [15, 38, 39], which does not seem to occur during the incubation adopted in the present study. Qualitatively, changes in the hydrolysis of LTA4 and on LTB4 content in CIA-arthritic and CIA-resistant, relatively to control, are parallel and positively related in all compartments under study, except in MB-ST and SO-PBMCs, pointing out these altered parameters as key differentiators of the healthy condition. On the other hand, quantitative differences of LTA4 hydrolysis and LTB4 content in CIA-arthritic and CIA-resistant distinguish these two situations between themselves. Comparing the changes in LTA4 hydrolysis and LTB4 content to control, it is noteworthy that they occur in the same sense in SF and MB-PBMCs of CIA-arthritic and CIA-resistant and in MB-ST of CIA-resistant, confirming, in these cases, the primordial role played by altered LTA4H in the biosynthesis of LTB4. Nevertheless, in the plasma and in SO-ST and SO-PBMCs from CIA-arthritic and CIA-resistant and in MB-ST of CIA-arthritic, the earlier mentioned correspondence between LTB4 and LTA4H is not found, indicating that setting of LTB4 content in these cases involves other steps in the biosynthesis and most probably in the degradation of LTB4 than the LTA4H activity. The changes in LTB4 content do not also have a unique sense in all samples from CIA-arthritic and CIA-resistant relatively to control, showing that levels of LTB4 vary through a compartment-dependent manner. Hashimoto et al. [40] reported the strong gene expression of LTB4 receptors, BLT1, and BLT2 in ST of arthritic patients. Mathis et al. [41] reported a reduction of incidence and severity of the disease, including protection against bone loss and cartilage injury, in mice knocked out for the BLT2 and with arthritis induced by autoantibodies. LTB4 in the plasma was higher during the active phase of rheumatoid arthritis than in the inactive phase or in healthy individuals [42]. Paradoxically, the present study shows that, in certain compartments (SO-PBMCs only in CIA-resistant and SO-ST and MB-PBMCs in CIA-arthritic and CIA-resistant), LTB4 levels, instead of being higher like in other samples examined, are relatively lower than control. These low levels of LTB4 in SO-ST in CIA-arthritic and CIA-resistant occur concomitantly with their increase in the MB-ST, suggesting a possible translocation of LTB4 from the cytosol to the membrane in response to inflammatory reaction. A possible diffusion of LTB4 to other cells and/or compartments during the inflammation could explain its low level in MB-PBMCs in CIA-arthritic and CIA-resistant and in SO-PBMCs in CIA-resistant. On the other hand, comparing CIA-arthritic and CIA-resistant in those compartments where they present different values for LTB4 or LTA4H (in MB-ST and SO-PBMCs for LTB4 and LTA4H and in SF for LTA4H), the highest levels of LTB4 content and LTA4H activity occur, respectively, in CIA-arthritic and CIA-resistant. This finding is coherent with the increase of LTB4 (proinflammatory) in CIA-arthritic, but it is a paradoxical occurrence in CIA-resistant, since increased LTA4 hydrolysis should lead to a higher LTB4 in CIA-resistant than in CIA-arthritic. Increased LTB4 in CIA-arthritic should contribute to the chronicity of inflammation and it occurs precisely in the MB-ST and SO-PBMCs, important targets of the disease. In addition to SF, LTA4H activity in CIA-resistant also increases in the same compartments, prominently in SO-PBMCs. Furthermore, Mendes et al. [7] reported that whereas basic aminopeptidase (APB) activity, which is known to be related to LTA4H activity [10, 13, 14], decreases in SO-PBMCs and SF, it remains unchanged in MB-ST of CIA-resistant. According to these findings, the LTB4 formation is inversely correlated with APB activity and its levels in these compartments would be comparatively more significant than the formation of LTB4 in the development of arthritis. Taken together, these results subsidize the hypothetical interrelation between the levels of LTB4 and LTA4H activity in CIA-arthritic, CIA-resistant, and control, as proposed in Figure 6.

In conclusion, we demonstrated that changes in LTB4 content and in LTA4H activity are qualitatively indistinguishable between CIA-arthritic and CIA-resistant, but such changes are compartment dependent and distinguish quantitatively these two groups between themselves and also these two groups from control. Compared with control, levels of LTB4 and LTA4H are only interrelated in SF and MB-PBMCs of CIA-arthritic and CIA-resistant, and in MB-ST of CIA-resistant, showing that pathophysiological levels of LTB4 are not only dependent on the activity of LTA4H.

## Figures and Tables

**Table 1:** LTB4 (ng/mL) in plasma, synovial fluid (SF), soluble (SO), and solubilized membrane-bound (MB) fractions from synovial tissue (ST) and peripheral blood mononuclear cells (PBMCs) from control, CIA-arthritic, and CIA-resistant rats.

Samples	Control	CIA-arthritic	CIA-resistant	ANOVA
PLASMA	0.1454 ± 0.0006^a^	0.2119 ± 0.0208^b^	0.2130 ± 0.0125^ab^	*P* = 0.0223
SF	0.0283 ± 0.0037^a^	0.0552 ± 0.0061^b^	0.0538 ± 0.0086^b^	*P* = 0.0214
SO-ST	0.4038 ± 0.0072^a^	0.1452 ± 0.0262^b^	0.1836 ± 0.0560^b^	*P* = 0.0090
MB-ST	0.0954 ± 0.0006^a^	0.1581 ± 0.0039^b^	0.1194 ± 0.0006^c^	*P* = 0.0007
SO-PBMCs	0.7163 ± 0.0141^a^	0.6975 ± 0.0168^a^	0.4368 ± 0.0139^b^	*P* < 0.0001
MB-PBMCs	0.3045 ± 0.0636^a^	0.0555 ± 0.0050^b^	0.0684 ± 0.0021^b^	*P* = 0.0051

Values are mean ± SEM. Number of animals = 3, values of each animal measured in duplicate. Student-Newman-Keuls, *P* < 0.05: comparison among control versus CIA-arthritic versus CIA-resistant in the same row (different letters indicate significant difference).

**Table 2:** LTA4H activity (U) in the plasma, synovial fluid (SF), soluble (SO), and solubilized membrane-bound (MB) fractions of synovial tissue (ST) and peripheral blood mononuclear cells (PBMCs) from control, CIA-arthritic, and CIA-resistant rats.

Samples	Control	CIA-arthritic	CIA-resistant	ANOVA
PLASMA	20.89 ± 0.77	28.92 ± 3.33	29.24 ± 3.77	*P* = 0.1540
SF	12.66 ± 0.86^a^	21.16 ± 1.34^b^	44.91 ± 3.00^c^	*P* < 0.0001
SO-ST	57.72 ± 8.20	38.81 ± 6.83	52.43 ± 4.61	*P* = 0.2023
MB-ST	13.85 ± 0.03^a^	0 ± 3.45^a^	56.29 ± 15.10^b^	*P* = 0.0060
SO-PBMCs	0 ± 12.48^a^	21.76 ± 10.01^b^	753.72 ± 5.23^c^	*P* < 0.0001
MB-PBMCs	142.30 ± 31.50^a^	57.42 ± 14.53^b^	48.86 ± 0.62^b^	*P* = 0.0306

Values are mean ± SEM. U = LTA4H unit = LTA4H activity in 1 mL sample that catalyzes the formation of 1 picomole of LTB4 per minute. Number of animals = 3, values of each animal measured in duplicate. Student-Newman-Keuls, *P* < 0.05: comparison among control versus CIA-arthritic versus CIA-resistant in the same row (different letters indicate significant difference).

**Table 3:** Changes in LTB4 and LTA4H in CIA-arthritic and CIA-resistant relatively to healthy control rats.

Samples	LTB4		LTA4H activity	
	CIA-arthritic	CIA-resistant	CIA-arthritic	CIA-resistant
Plasma	↑	↑	=	=
Synovial fluid	↑	↑	↑	↑
Soluble fraction from synovial tissue	↓	↓	=	=
Solubilized membrane-bound fraction from synovial tissue	↑	↑	=	↑
Soluble fraction from PBMCs	=	↓	↑	↑
Solubilized membrane-bound fraction from PBMCs	↓	↓	↓	↓

Endogenous LTB4 content and LTA4H activity assessed by EIA. ( = ) no difference; (↓) decrease; (↑) increase.
